# The heterogeneous functional architecture of the posteromedial cortex is associated with selective functional connectivity differences in Alzheimer's disease

**DOI:** 10.1002/hbm.24894

**Published:** 2019-12-19

**Authors:** Wasim Khan, Ali Amad, Vincent Giampietro, Emilio Werden, Sara De Simoni, Jonathan O'Muircheartaigh, Eric Westman, Owen O'Daly, Steve C. R. Williams, Amy Brodtmann, for the Alzheimer's Disease Neuroimaging Initiative

**Affiliations:** ^1^ The Florey Institute for Neuroscience and Mental Health University of Melbourne Melbourne Victoria Australia; ^2^ Department of Neuroimaging Institute of Psychiatry, Psychology, and Neuroscience (IoPPN), King's College London London UK; ^3^ Univ Lille Nord de France, CHRU de Lille Lille France; ^4^ Computational, Cognitive and Clinical Neuroimaging Laboratory Imperial College London, Division of Brain Sciences, Hammersmith Hospital London UK; ^5^ Department of Neurobiology Care Sciences and Society, Karolinska Institute Stockholm Sweden; ^6^ NIHR Biomedical Research Centre for Mental Health King's College London London UK; ^7^ NIHR Biomedical Research Unit for Dementia King's College London London UK; ^8^ Austin Health, Heidelberg Melbourne Victoria Australia; ^9^ Eastern Clinical Research Unit Monash University, Box Hill Hospital Melbourne Victoria Australia; ^10^ Department of Forensic and Neurodevelopmental Sciences Institute of Psychiatry, Psychology, and Neuroscience (IoPPN), King's College London London UK; ^11^ Department of Perinatal Imaging and Health St. Thomas' Hospital, King's College London London UK; ^12^ MRC Centre for Neurodevelopmental Disorders King's College London London UK

**Keywords:** Alzheimer disease, fMRI, magnetic resonance imaging, multivariate analysis, posterior cingulate cortex, precuneus

## Abstract

The posteromedial cortex (PMC) is a key region involved in the development and progression of Alzheimer's disease (AD). Previous studies have demonstrated a heterogenous functional architecture of the region that is composed of discrete functional modules reflecting a complex pattern of functional connectivity. However, little is understood about the mechanisms underpinning this complex network architecture in neurodegenerative disease, and the differential vulnerability of connectivity‐based subdivisions in the PMC to AD pathogenesis. Using a data‐driven approach, we applied a constrained independent component analysis (ICA) on healthy adults from the Human Connectome Project to characterise the local functional connectivity patterns within the PMC, and its unique whole‐brain functional connectivity. These distinct connectivity profiles were subsequently quantified in the Alzheimer's Disease Neuroimaging Initiative study, to examine functional connectivity differences in AD patients and cognitively normal (CN) participants, as well as the entire AD pathological spectrum. Our findings revealed decreased functional connectivity in the anterior precuneus, dorsal posterior cingulate cortex (PCC), and the central precuneus in AD patients compared to CN participants. Functional abnormalities in the dorsal PCC and central precuneus were also related to amyloid burden and volumetric hippocampal loss. Across the entire AD spectrum, functional connectivity of the central precuneus was associated with disease severity and specific deficits in memory and executive function. These findings provide new evidence showing that the PMC is selectively impacted in AD, with prominent network failures of the dorsal PCC and central precuneus underpinning the neurodegenerative and cognitive dysfunctions associated with the disease.

## 1 INTRODUCTION

Alzheimer's disease (AD) is a progressive neurodegenerative disorder characterised by a decline in memory and cognitive functions. The disease is related to the pathological accumulation of aggregated amyloid depositions and hyperphosphorylation of structural proteins which lead to metabolic alterations, functional loss, and structural changes in the brain. Convergent evidence across neuroscience disciplines suggests that proteinopathies progress trans‐synaptically along brain networks, with neuronal dysfunction topographically spreading from a region of focal onset to non‐adjacent regions in a predictable pattern manifesting over several years (Greicius & Kimmel, 2012; Liu et al., 2012). The location and distribution of pathogenic processes such as the accumulation of amyloid deposits has been consistently mapped to a network of heteromodal regions collectively known as the default mode network (DMN; Buckner et al., 2005).

Some of the earliest and consistent pathological changes observed in AD are evident in the posteromedial cortex (PMC)—an integrated hub region important for episodic memory encoding and retrieval (Greicius, Srivastava, Reiss, & Menon, 2004; K. Wang et al., 2007). Large decrements in glucose metabolism of the PMC and a vulnerability to amyloid pathology are consistent early features of AD which are known to manifest prior to the onset of clinical symptoms (Minoshima et al., 1997; Mintun et al., 2006). In the later stages of AD pathogenesis, a disruption to connections between the PMC and large‐scale memory and visual networks can also be observed (H. Y. Zhang et al., 2009). This suggests that an accurate characterisation of PMC function is of vital importance to the understanding of AD development and progression. However, little is known about the pattern of PMC functional connectivity with distributed large‐scale brain networks across different stages of the AD pathological spectrum.

Previous anatomical studies have demonstrated that the PMC consists of highly diverse cytoarchitectonics and is functionally heterogenous (Margulies et al., 2009; Parvizi, Van Hoesen, Buckwalter, & Damasio, 2006; Vogt & Laureys, 2005). Yet, the PMC is often treated as having a homogenous functional architecture in studies of the DMN, despite its diverse patterns of functional connectivity (Y. Zhang et al., 2014). Recent work characterising the functional architecture of the PMC has shown that it consists of several different functional subdivisions that are associated with multiple large‐scale brain networks at rest (Kernbach et al., 2018; Leech, Braga, & Sharp, 2012; S. Zhang & Li, 2012). In particular, the posterior cingulate cortex (PCC), which lies in the medial part of the inferior parietal lobe, exhibits distinct cytoarchitectonics with functional separation into dorsal and ventral areas (Leech, Kamourieh, Beckmann, & Sharp, 2011; Vogt, Vogt, & Laureys, 2006). This dorsal region of the PCC demonstrates strong connectivity with the DMN and other large‐scale networks, including the frontoparietal network involved in executive control and the salience network for attention, thus implicating its role in modulating global network metastability (Hellyer, Scott, Shanahan, Sharp, & Leech, 2015; Leech et al., 2012). In contrast, the ventral region of the PCC is highly integrated within the DMN, particularly with key medial prefrontal and temporal nodes and is understood to be involved in internally directed cognition, such as memory retrieval and planning (Dastjerdi et al., 2011; Leech et al., 2012). As a result, studies have suggested that the PMC plays a central associative role across a wide‐spectrum of integrated functions with evidence of its involvement in sensorimotor processing, cognitive functioning, and the processing of visual information (Hutchison, Culham, Flanagan, Everling, & Gallivan, 2015). However, only a handful of studies have examined the different functional subdivisions of the PMC in AD (Cauda et al., 2010; Xia et al., 2014). Furthermore, no studies have addressed, to the best of our knowledge the susceptibility of these subdivisions across the entire AD spectrum as well as the relationship between PMC subdivisions and other well‐established disease markers of AD pathology.

A few resting‐state fMRI (rsfMRI) studies in AD have parcellated the PMC to examine its intrinsic functional architecture, however most have used a priori defined cortical seed regions to characterise its complex functional neuroanatomy (Cauda et al., 2010; Dillen et al., 2016; Margulies et al., 2009; Wu et al., 2016). Recent work has highlighted the advantages of connectivity‐based parcellation methods for a more detailed insight into the organisation of regional specialisation of brain regions (Eickhoff, Thirion, Varoquaux, & Bzdok, 2015; Thirion, Varoquaux, Dohmatob, & Poline, 2014).

Here, we used high‐resolution rsfMRI data from the Human Connectome Project (HCP) to fractionate the PMC into its subdivisions using a constrained independent component analysis (ICA) method and characterise its unique patterns of functional connectivity with large‐scale brain networks. Subsequently, these detailed maps of the PMC were used to compare functional connectivity differences in AD using the publicly available and widely phenotyped Alzheimer's Disease Neuroimaging Initiative (ADNI) study. Specifically, the aim of this study was to examine functional connectivity changes in the different subdivisions of the PMC across the AD spectrum (*N* = 155), ranging from cognitively normal (CN) participants and participants with subjective memory complaints (SMC) through to those with mild cognitive impairment (MCI), and AD. We first characterised the functional connectivity of the PMC by examining disease‐specific changes in AD patients compared to CN participants. The functional connectivity of PMC subdivisions that were disrupted in AD were subsequently tested for their association with amyloid burden and hippocampal volume measurements. Furthermore, we hypothesised that brain networks of PMC subdivisions that were strongly implicated in cognition would be associated with specific deficits in memory and executive function.

## 2 MATERIALS AND METHODS

For this study, data was obtained from HCP (Van Essen et al., 2012; http://www.humanconnectome.org/) for defining PMC subdivisions and their functional connectivity patterns in healthy unaffected young adults. These detailed functional maps and results were later used to assess functional connectivity patterns in AD patients and CN participants from the ADNI database (http://adni.loni.usc.edu/). For the purposes of simplicity, we will describe these different datasets in the same order as the workflow from our analysis pipeline.

### 2.1 Human Connectome Project

#### 2.1.1 Data and preprocessing

rsfMRI data for 100 unrelated healthy adult participants (age range: 22–36 years; 46 males) were obtained from the HCP S1200 data release. Participants had no documented history of mental illness, neurological disorder, or physical illness with known impact upon brain functioning. This cohort of participants was selected such that there were no related participants within the cohort due to concerns over the heritability of neural features (Glahn et al., 2010). Data were acquired on a Siemens Skyra 3T scanner housed at Washington University in St. Louis (TR = 720 ms, TE = 33.1 ms, spatial resolution = 2 × 2 × 2 mm^3^), collected in four separate 15‐min runs on two different days (two per day). Each rsfMRI run consisted of 1,200 volumes which totalled to 4,800 volumes (over the four runs). Quality assurance and quality control procedures of HCP for rsfMRI data have been described previously (Marcus et al., 2013). The data we obtained had been minimally preprocessed by HCP (Fischl, 2012; Glasser et al., 2013; Jenkinson, Bannister, Brady, & Smith, 2002; Jenkinson, Beckmann, Behrens, Woolrich, & Smith, 2012), as well as denoised to remove non‐neural spatiotemporal components (Griffanti et al., 2014; Salimi‐Khorshidi et al., 2014) and high‐pass filtered (Satterthwaite et al., 2013).

#### 2.1.2 Defining PMC functional subdivisions

**Figure 1:**
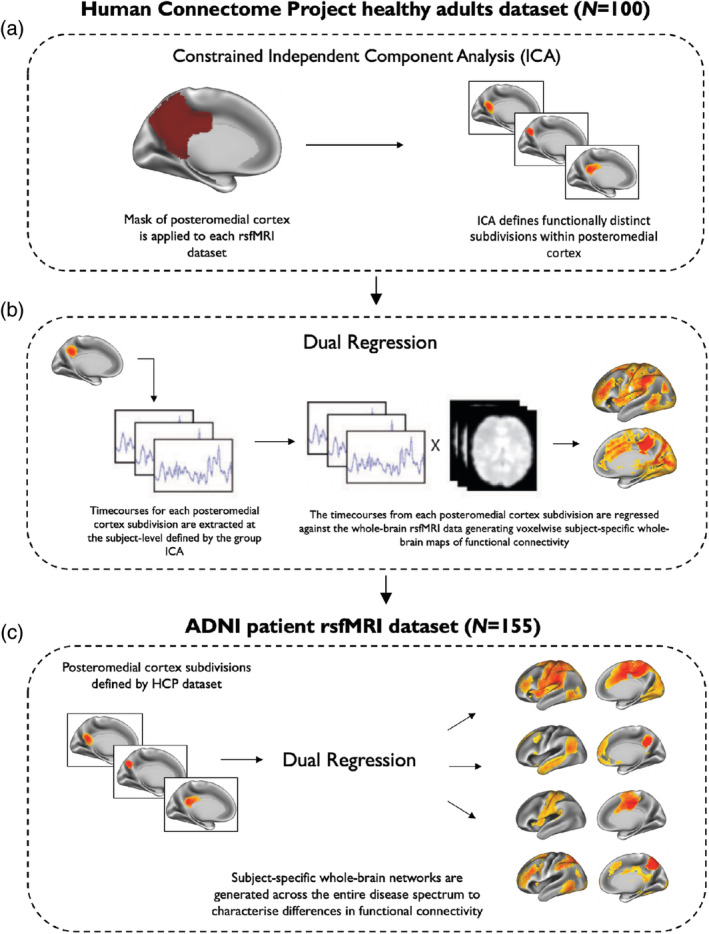
A schematic representation of the major steps involved in the functional connectivity analysis of the posteromedial cortex. (a) An ICA approach was used to fractionate subdivisions of the posteromedial cortex (PMC) by constraining the analysis to voxels within a pre‐defined PMC mask in the HCP dataset. (b) In the same HCP dataset, a dual‐regression analysis was used to define the functional connectivity patterns of each PMC subdivision by correlating its activity with voxels in the rest of the brain. (c) PMC subdivisions defined in the HCP dataset were used to characterise whole‐brain functional connectivity of brain networks identified in the ADNI patient dataset (*N* = 155). This dataset included cognitively normal (CN) participants, participants with subjective memory complaints (SMC), early mild cognitive impairment (MCI) participants, late MCI participants, and patients with Alzheimer's disease (AD). The brain networks shown here in (c) were constrained to voxels of the same brain networks identified in the HCP dataset, shown in (b)

A constrained ICA was performed using the minimally preprocessed HCP datasets in the FMRIB Software Library (FSL; 5.0.11) using the MELODIC tool (Beckmann, DeLuca, Devlin, & Smith, 2005). A temporal concatenation group ICA was constrained to extract components within a PMC mask, defined a priori using the Harvard‐Oxford probabilistic atlas (Figure 1a). The PMC was constructed by selecting PCC and precuneus regions with a voxel probability threshold greater than 20% signal intensity. rsfMRI data within the mask of the PMC were spatially smoothed using an 8 mm full width at half maximum Gaussian (FWHM) kernel following masking. In order to determine the ideal number of ICA components to decompose the PMC into its distinct functional subdivisions, we performed a reproducibility analysis to assess the procedures trade‐off between granularity and noise using the mICA toolbox (Moher Alsady, Blessing, & Beissner, 2016). Reproducibility analyses were performed for an ICA dimensionality range of 2–20 ICA components and showed that 10 ICA components were best representative of the underlying HCP data. Consequently, MELODIC was run to extract 10 ICA components for the PMC. Further details regarding the reproducibility analysis are provided in the Supporting Information Methods section.

#### 2.1.3 Characterising cortical functional connectivity of PMC subdivisions

Functional connectivity analysis was performed using a variant of the dual regression approach in FSL (Beckmann, Mackay, Filippini, & Smith, 2009; Filippini et al., 2009). The dual regression approach was primarily chosen to construct spatial maps that contain voxelwise information about the spatial location and magnitude of functional connectivity at the individual subject level with corresponding temporal dynamics contained within each PMC subdivision. Dual regression is a tool that utilises individual ICA components as templates to identify the corresponding functional connectivity maps of each participant (Nickerson, Smith, Öngür, & Beckmann, 2017). In accordance with previous work (Bonnelle et al., 2011; Leech et al., 2012; De Simoni et al., 2018), dual regression was used in the present study to obtain a voxelwise measure of functional connectivity between each voxel in the brain and the decomposed ICA signal of the PMC. This resulted in whole‐brain networks of the PMC corresponding to the subdivisions identified with ICA. To define the functional connectivity patterns of each PMC subdivision in the HCP dataset using this variant of dual regression, a general‐linear model was applied in two steps. First, all unthresholded ICA spatial maps of the PMC subdivisions were linearly regressed against whole‐brain rsfMRI data (spatial regression), resulting in subject‐specific timecourses for each ICA spatial map (i.e., each PMC subdivision). This step served to generate a subject‐specific timecourse for each spatial map of the ICA while controlling for the variance explained by the other spatial maps. Second, these timecourses were variance normalised and linearly regressed against whole‐brain rsfMRI data in a separate general‐linear model. In this step, timeseries were converted to subject‐specific whole‐brain spatial maps of the corresponding ICA component reflecting network coherence for each PMC subdivision (Figure 1b). Group average maps were calculated using a general‐linear model (Figure 1c). To account for multiple comparisons, the TFCE method was used (Smith & Nichols, 2009) with 5,000 permutations.

### 2.2 ADNI patient dataset

Data used for this study was obtained from the ADNI database (http://adni.loni.usc.edu/). ADNI is a multi‐centre longitudinal biomarker study that has enrolled over 1,500 CN participants, people with early or late stages of MCI, and patients with early AD (http://www.adni-info.org). ADNI was launched in 2003 as a public‐private partnership, led by Principal Investigator Michael W. Weiner, MD and was approved by the institutional review board and ethics committees of participating institutions. Written informed consent was obtained according to the Declaration of Helsinki from all participants or their next of kin. For up‐to‐date information, see http://www.adni-info.org.

#### 2.2.1 Participants

All participants were downloaded from the ADNI‐2 database (June 2018). Baseline scans were identified from all images that had undergone quality control implemented by the Mayo Clinic (*N* = 827; Jack et al., 2015). We selected all first available scans for each participant as their baseline scan (*N* = 225). Based on our inclusion criteria, several participants were excluded (*N* = 53) to ensure that we only retained data of a reasonable quality. This included any scans with motion parameters exceeding 1.5 mm of translation and/or rotation (*N* = 11), scans with an image quality rating >3 (image quality rated as: 1 = excellent; 2 = good; 3 = fair; 4 = poor; *N* = 36), presence of microhaemorrhages or cysts (*N* = 3), or any other uncertainties in diagnosis (*N* = 3). Of the remaining 172 participants, if amyloid imaging or *APOE* genotyping was unavailable (*N* = 17), these participants were not included for further analysis. PET amyloid imaging was performed using Florbetapir. A measure of amyloid burden was calculated from frontal, cingulate, parietal, and temporal regions and was averaged and divided by a whole cerebellum reference region to create a standardised uptake value ratio. A threshold of 1.11 was used to define amyloid positivity. This has been described in detail previously (Landau et al., 2013). Hippocampal volumes were calculated using FreeSurfer (version 6.0; http://surfer.nmr.mgh.harvard.edu/***; Fischl, 2012).

A full description of the structural and functional image preprocessing steps for the ADNI study are provided in the Supporting Information Methods section. The final dataset of participants used in this study is summarised in Table 1. A complete list of participant scans used in this study is provided in Supporting Information Table S1.

**Table 1:** Demographic characteristics and metadata of the ADNI rsfMRI dataset (*N* = 155)

	CN (*N* = 34)	SMC (*N* = 24)	EMCI (*N* = 43)	LMCI (*N* = 31)	AD (*N* = 23)	*P*‐value
Baseline age (years)	75.3 ± 6.3	71.9 ± 5.3	71.1 ± 6.9	71.2 ± 7.7	72.9 ± 7.7	.062
Sex (male %)	14 (41)	10 (42)	17 (40)	20 (65)	12 (52)	.20
Years of education	16.1 ± 2.0	16.7 ± 2.9	15.8 ± 2.8	16.6 ± 2.6	15.6 ± 2.7	.28
*APOE* ε4 carriers (%)	11 (32)	8 (33)	22 (51)	14 (45)	18 (78)	**.01**
CDR sum‐of‐boxes	0.06 ± 0.2^a^^,^ b ^,^ c	0.04 ± 0.1^a^^,^ b ^,^ c	1.4 ± 1.0^a^^,^ b ^,^ e	1.7 ± 1.0^c^^,^ d ^,^ e	4.5 ± 1.2^a^^,^ b ^,^ d ^,^ e	**<.001**
MMSE	28.9 ± 1.1^b^^,^ c	29.1 ± 0.9^b^^,^ c	28.3 ± 1.7^c^	27.5 ± 1.5^c^^,^ d ^,^ e	22.3 ± 2.5^a^^,^ b ^,^ d ^,^ e	**<.001**
ADAS‐Cog11	5.6 ± 2.5^a^^,^ b ^,^ c	5.6 ± 2.3^b^^,^ c	7.8 ± 3.3^b^^,^ c ^,^ d	10.9 ± 4.1^a^^,^ c ^,^ d ^,^ e	24.3 ± 7.8^a^^,^ b ^,^ d ^,^ e	**<.001**
RAVLT forgetting (%)	39.1 ± 24.4^b^^,^ c	37.7 ± 22.4^b^^,^ c	54.7 ± 29.1^c^	67.6 ± 25.9^c^^,^ d ^,^ e	95.5 ± 10.4^a^^,^ b ^,^ d ^,^ e	**<.001**
Trail making test B	89 ± 64^c^	80 ± 42^e^	100 ± 48^c^	112 ± 65^c^	209 ± 86^a^^,^ b ^,^ d ^,^ e	**<.001**
Amyloid Florbetapir SUVR^g^	1.15 ± 0.20^c^	1.13 ± 0.18^c^	1.21 ± 0.21^c^	1.26 ± 0.25^c^	1.45 ± 0.18^a^^,^ b ^,^ d ^,^ e	**<.001**
Hippocampal volume (ml)^f^	7.6 ± 0.84^c^	7.7 ± 1.1^e^	7.4 ± 0.9^c^	7.2 ± 1.3	6.1 ± 1.1^a^^,^ d ^,^ e	**<.001**
Framewise displacement	0.15 ± 0.09	0.17 ± 0.09	0.14 ± 0.07	0.13 ± 0.05	0.13 ± 0.06	.66

*Note:* Results are displayed as mean ± *SD*. A Kruskal–Wallis rank sum test was used for comparison of group differences in continuous variables. Categorical variables were inspected for group differences using a Fisher's exact test with *p*‐values generated using 2000 Monte Carlo simulations.

Abbreviations: AD, Alzheimer's disease; ADAS‐cog11, 11‐item Alzheimer's disease assessment scale‐cognitive subscale; CDR, clinical dementia rating scale sum‐of‐boxes; CN, cognitively normal; EMCI, early mild cognitive impairment; LMCI, late mild cognitive impairment; MMSE, mini‐mental‐state examination; RAVLT, Rey auditory verbal learning test; SMC, subjective memory complaints; SUVR, standardised uptake value ratio.

a Significant compared to EMCI participants.

b Significant compared to LMCI participants.

c Significant compared to AD patients.

d Significant compared to CN participants.

e Significant compared to SMC participants.

f Fourteen subjects were not considered due to poor FreeSurfer segmentations (SMC = 3, EMCI = 1, LMCI = 7, AD = 3).

g Fifteen subjects did not have available amyloid PET imaging (CN = 8, SMC = 2, LMCI = 4, AD = 1).

#### 2.2.2 Assessment of memory performance and executive functioning

As a measure of memory performance, we used mini‐mental‐state examination (MMSE) scores and the 11‐item Alzheimer's disease assessment scale‐cognitive subscale (ADAS‐Cog11) scores. Neuropsychological measures of verbal memory and executive function included Rey auditory verbal learning test (RAVLT) percentage forgetting scores and trail making test B scores, respectively.

#### 2.2.3 Examining PMC functional connectivity patterns in AD patients and healthy controls

The 10 ICA spatial maps of the PMC subdivisions defined in the HCP dataset were used to characterise whole‐brain PMC functional connectivity patterns in the ADNI dataset (*N* = 155). This was performed using the same dual regression procedure described above. Whole‐brain maps of PMC functional connectivity were constrained to voxels identified within the same corresponding whole‐brain map from the HCP cohort (Figure 1c). These functional maps were subsequently transformed back to their original native space. This resulted in functional maps that contained voxelwise information about the spatial location and magnitude of functional connectivity at the individual subject level (Filippini et al., 2009; D. T. Jones et al., 2016). The average beta of these spatial maps across all voxels was extracted as a final measure of functional connectivity. This approach was preferred over voxelwise statistical comparisons to avoid the potentially huge multiple comparison penalty associated with comparing several PMC networks across multiple groups. Summary metrics of these brain networks also provided the opportunity to extensively investigate their relationship with disease markers of AD pathology.

#### 2.2.4 Statistical analysis

All subsequent analyses were performed using the R statistical software environment (http://www.r-project.org; version 3.5.1; The R Core Team, 2018). Conditions for meeting normality assumptions were tested using QQ‐plots, the distribution of residuals and the absence of multicollinearity. A Kruskal–Wallis rank sum test was used to compare continuous demographic and cognitive measures between groups and pairwise group differences were inspected using a pairwise Wilcoxon rank sum test with a Bonferroni adjustment. Categorical variables were compared using a Fisher's exact test. PMC brain networks that were not normally distributed were transformed using a *Z*‐family of distributions (Chou, Polansky, & Mason, 1998; Johnson, 1949), after which no deviation from a normal distribution was observed via a Shapiro–Wilk normality test.

PMC functional connectivity differences were assessed between AD patients and healthy controls using a multivariate linear regression. Two different models were constructed: the first using age, sex, years of education and framewise displacement as covariates and the second additionally correcting for *APOE* ε4 genotype. Statistical significance of regression models was assessed using Type II multivariate analysis of variance (MANOVA) tests and the Pillai test statistic. Next, we tested the association of PMC brain networks found to be disrupted in AD with amyloid burden and hippocampal volume. These associations were tested using linear stepwise regression models in the R MASS package (Venables & Ripley, 2002) Models included predictors such as age, sex, years of education, and *APOE* ε4 genotype. Stepwise model selection was performed using the Akaike Information Criterion (AIC) using both backward and forward selection of predictors. For comparisons with hippocampal volume, intracranial volume measurements were always included as a predictor in the final model.

To investigate the effect of PMC functional connectivity on cognition, we applied linear regression models on all participants at different stages of the AD pathological spectrum (*N* = 155; Table 1). Firstly, we tested the relationship between specific PMC brain networks that were found to be abnormal in AD with memory performance (i.e., MMSE) and clinical measures of disease severity (i.e., ADAS‐Cog11). In additional models, we also compared disrupted PMC brain networks with executive functioning, using RAVLT percentage forgetting scores, and verbal memory measured using trail making test B scores. Regression models were constructed in a similar fashion as described above.

All analyses were corrected for multiple comparisons using the Bonferroni adjustment. Only results that survived multiple comparison correction with an alpha threshold of *α* = 0.05 were reported in this study.

## 3 RESULTS

### 3.1 ADNI demographics

A total of 155 participants were selected for this study, consisting of CN participants (*N* = 34), participants with SMC (*N* = 24), early MCI participants (*N* = 43), late MCI participants (*N* = 31), and patients with AD (*N* = 23; Table 1). *APOE* ε4 carriers were significantly greater in proportion in AD patients, followed by early and late MCI participants (*p* = .01). Overall, AD patients and MCI participants demonstrated greater functional and cognitive impairments compared to CN participants on several clinical and neuropsychological tests (*p* = <.001). No significant differences were observed for sex distribution, years of education, and maximum framewise displacement of rsfMRI scans. We also did not observe any significant correlations between framewise displacement and PMC functional connectivity.

### 3.2 PMC fractionation reveals distinct functional subdivisions

**Figure 2:**
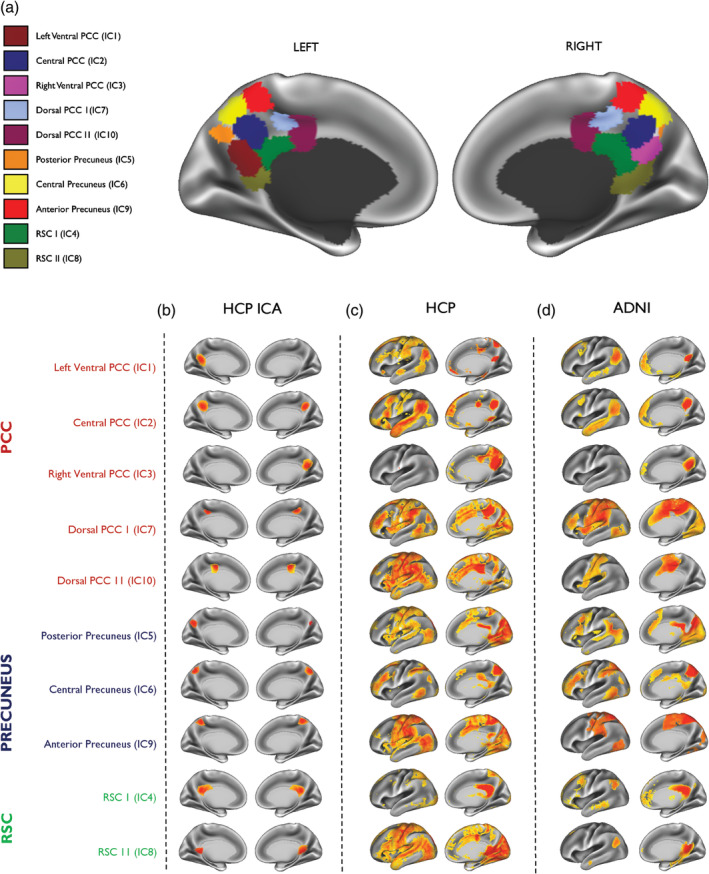
Subdivisions of the posteromedial cortex and their associated brain networks in the HCP cohort and ADNI patient dataset. (a) A parcellation map showing the location of all subdivisions defined in the posteromedial cortex (PMC) using the HCP dataset. (b) The location of each PMC subdivision is shown separately on the far left. Each subdivision (displayed as left and right medial hemispheres) is numbered by its ICA component and highlighted as anatomically representing the posterior cingulate cortex (PCC) in red, precuneus in blue and the retrosplenial cortex (RSC) in green. The whole‐brain network (displayed as left lateral and right medial hemisphere) of the corresponding PMC subdivision is shown for (c) the HCP dataset and (d) the ADNI patient dataset (*N* = 155). Warmer colours indicate areas of high functional connectivity. All maps are thresholded at *p* < .05 and are family‐wise‐error corrected for multiple comparisons

The ICA analysis from the HCP cohort identified 10 functionally distinct and spatially overlapping subdivisions of the PMC. These subdivisions can be observed on a functional parcellation map of the PMC shown in Figure 2a. Each PMC subdivision is also illustrated separately in Figure 2b. To ensure that our PMC fractionation was representative of the underlying HCP data and not an artefact of the number of components chosen for ICA decomposition, we performed a reproducibility analysis to select the ideal number of ICA components for decomposing the PMC signal. Results of this analysis demonstrated that 10 ICA components were ideal for fractionating the PMC (see Supporting Information Figure S1).

**Figure 3:**
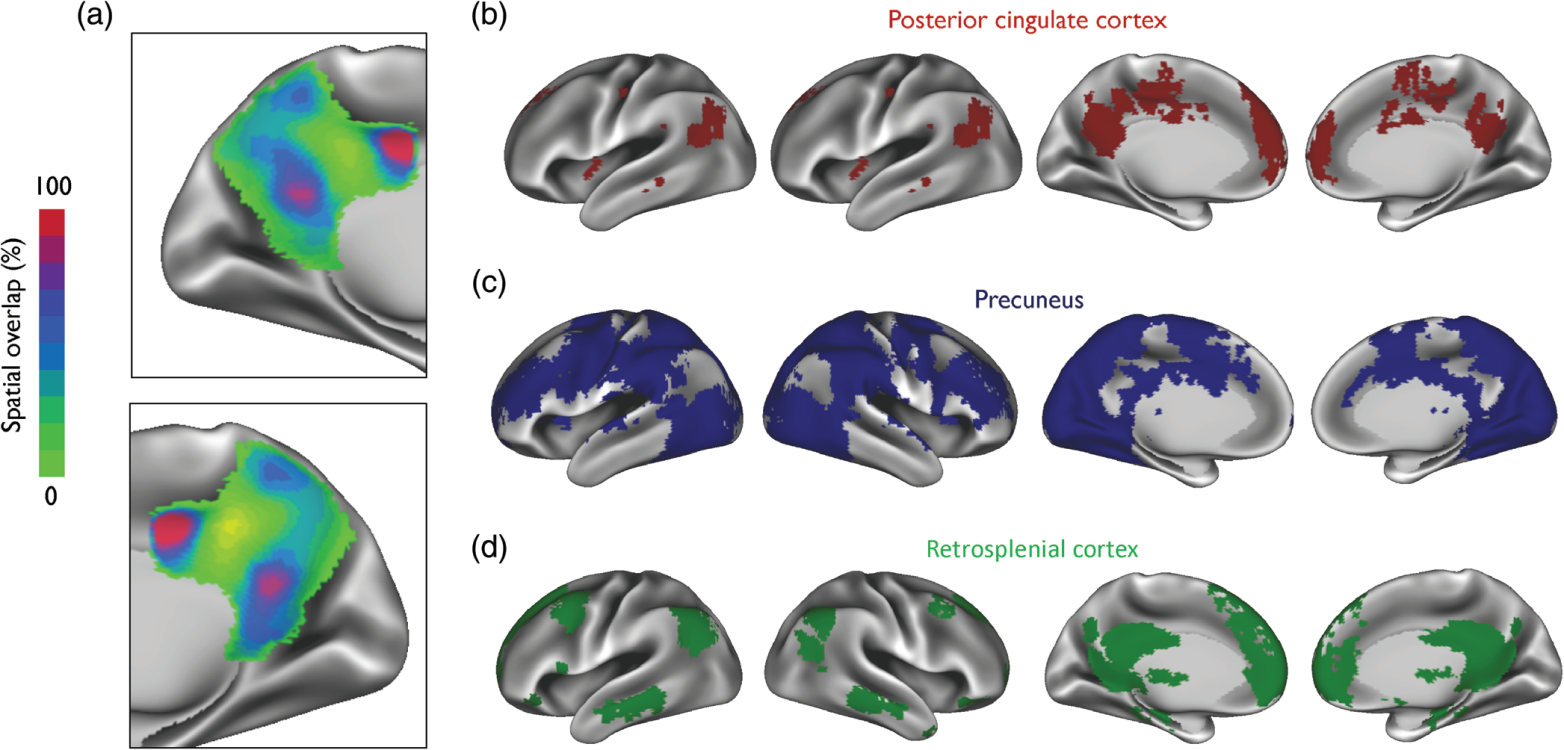
A spatial overlay map of posteromedial cortex functional subdivisions and its corresponding brain networks. (a) The overlap between all 10 subdivisions of the posteromedial cortex (PMC) is shown demonstrating the greatest overlap in the dorsal region of the posterior cingulate cortex (PCC), the ventral PCC and parts of the retrosplenial cortex (RSC). Also shown are spatial overlay masks of all brain networks originating from (b) the PCC, (c) the precuneus, and (d) the RSC. Maps are displayed as left and right lateral and medial hemispheres

As illustrated in Figure 2a,b, five subdivisions were found to be located in the PCC, primarily in the ventral, dorsal, and anterior‐dorsal parts. Three subdivisions were located in the anterior, central and posterior parts of the precuneus. Two subdivisions were found to be located in the RSC. Although PMC subdivisions shared a relatively low spatial similarity overall (*r* = .06–.113), the highest spatial overlap was found between the dorsal and ventral parts of the PMC (*r* = .113; Figure 3a). This suggests that, despite some spatial overlap, the ICA results produced maps of considerable granularity and spatial separation.

### 3.3 Functional subdivisions of the PMC are connected to several distinct brain networks

Next, we investigated whether signals from the different PMC subdivisions correlated with activity in the rest of the brain. Subdivisions of the PMC and their associated brain networks are shown in Figure 2. Our findings showed a complex functional heterogeneity of the PMC where different subdivisions were found to be associated with several distinct brain networks (Figure 2c,d). The three subdivisions (IC5, IC6 and IC9) located within the precuneus revealed a distributed pattern of functional connectivity and included areas such as the frontal pole, supramarginal gyrus, temporal gyrus, occipital cortex, and occipital fusiform gyrus. For the five subdivisions of the PCC (IC1, IC2, IC3, IC7 and IC10), we found a more organised pattern of functional connectivity. Functional networks associated with IC1, IC2 and IC3 of the PCC were more DMN‐like in appearance, whereas IC3 and IC7 resembled salience and frontoparietal networks covering parts of the inferior parietal regions, dorsolateral prefrontal cortex and pre‐supplementary motor area. For the RSC, the two subdivisions (IC4 and IC8) showed similar patterns of functional connectivity.

We further generated spatial overlay masks of all the brain networks arising from the precuneus (Figure 3b), the PCC (Figure 3c), and the RSC (Figure 3d). These were not used to compare functional connectivity differences in our study, but rather to show areas of the brain that were functionally common within anatomical regions of the PMC. Overlay masks of the precuneus reveal that more areas of the brain are functionally correlated with its different functional subdivisions. For the precuneus and RSC, overlay masks appear to be topographically similar to the DMN.

### 3.4 Functional connectivity of the PMC is reduced in AD

We compared the functional connectivity differences in PMC activity between AD patients (*N* = 23) and CN participants (*N* = 34). Results are described from two models, one corrected for age, sex, years of education and framewise displacement and the second additionally correcting for *APOE* ε4 genotype. These results are shown in Table 2. Comparisons were performed for the functional connectivity of all PMC subdivisions as no a priori hypotheses about specific network changes were postulated. The overall multivariate regression model was statistically significant (Pillai test statistic = 0.32; *p* = .002) and remained significant when additionally correcting for *APOE* ε4 genotype.

**Table 2:** PMC functional connectivity differences in AD patients and CN participants

	*t*	Cohen's *d*	*P*‐value^a^	*P*‐value^b^
PCC				
Left ventral PCC (IC 1)	−1.83	−.49	.07	.05
Central PCC (IC 2)	−1.76	−.48	.08	.07
Right ventral PCC (IC 3)	0.13	.04	.90	.99
Dorsal PCC I (IC 7)	−2.93	−.79	.004	.003
Dorsal PCC II (IC 10)	−1.06	−.29	.29	.36
Precuneus				
Posterior precuneus (IC 5)	−1.45	−.39	.15	.35
Central precuneus (IC 6)	−3.20	−.86	<.001	<.001
Anterior precuneus (IC 9)	−2.68	−.72	.008	.019
RSC				
RSC I (IC 4)	−0.68	−.18	.50	.69
RSC II (IC 8)	0.92	.25	.36	.13

*Note:* Results have been corrected for multiple comparisons using the Bonferroni method.

Abbreviations: AD, Alzheimer's disease; CN, cognitively normal participants; IC, independent component; PCC, posterior cingulate cortex; PMC, posteromedial cortex; RSC, retrosplenial cortex.

a Corrected for age, sex, years of education, and framewise displacement (MANOVA Pillai test statistic = 0.321; *p* = .002).

b Corrected for age, sex, years of education, *APOE* ε4 genotype, and framewise displacement (MANOVA Pillai test statistic = 0.329; *p* = .002).

**Figure 4:**
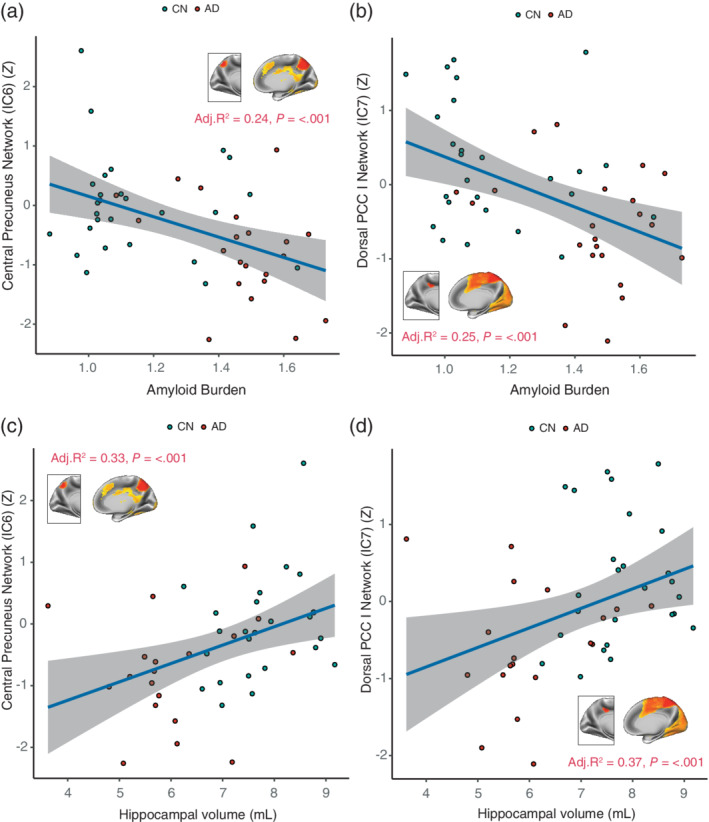
The relationship of the central precuneus and dorsal PCC networks with amyloid burden and hippocampal volume. Results are displayed for AD patients (*N* = 23) and CN participants (*N* = 34) in the ADNI dataset. Functional connectivity of (a) the central precuneus network, and (b) the dorsal PCC plotted versus PET Florbetapir measures of amyloid burden. Similar plots are illustrated for (c) the central precuneus network, and (d) the dorsal PCC network against FreeSurfer derived measures of hippocampal volume. The variance explained by each of the models (Adj. *R* ^2^) and *p*‐values are displayed inset. Individual data points, regression lines and 95% CIs (grey bands) are displayed for each plot. Covariates considered in regression models included age, gender, years of education and *APOE* ε4 genotype. Models of hippocampal volume were corrected for intracranial volume measurements. AD, Alzheimer's disease; CN, cognitively normal; PCC, posterior cingulate cortex

Functional networks associated with two PCC subdivisions demonstrated reduced functional connectivity patterns compared to CN participants. This included the brain network of the left ventral PCC (IC3; *t* = −1.83; Cohen's *d* = −.49; *p* = .05) and the dorsal PCC (*t* = −2.93; Cohen's *d* = −.79; *p* = .003). For the precuneus, functional networks of the central precuneus subdivision (*t* = −3.20; Cohen's *d* = −.86; *P* = < .001) and the anterior precuneus subdivision (*t* = −2.68; Cohen's *d* = −.72; *p* = .019) were significantly reduced in AD patients. No functional connectivity differences were observed for subdivisions of the RSC. We also did not find any significant increases in functional connectivity. Linear regression analyses were performed using these brain networks that were disrupted in AD patients to determine their association with amyloid burden and hippocampal volume. Results revealed that decreased functional connectivity of the dorsal PCC, and central precuneus were associated with greater levels of amyloid burden and lower hippocampal volumes (Figure 4).

### 3.5 Central precuneus functional connectivity is associated with clinical disease progression, memory deficits and executive dysfunction

The central precuneus was strongly decreased in AD patients and has been previously implicated to play an integrated cognitive/associative role in the brain (Margulies et al., 2009). Therefore, we sought to determine whether aberrant functional connectivity of this PMC subdivision would be associated with disease severity and specific deficits in memory. Since no prior hypotheses were established regarding the different pathophysiological profiles of AD, we did not stratify participants on the basis of amyloid status (i.e., amyloid positive vs. amyloid negative). Instead, participants across the entire disease spectrum were included (*N* = 155; Table 1), ranging from CN participants and participants with SMC, through to participants with early and late stages of MCI, and finally patients diagnosed with AD.

**Figure 5:**
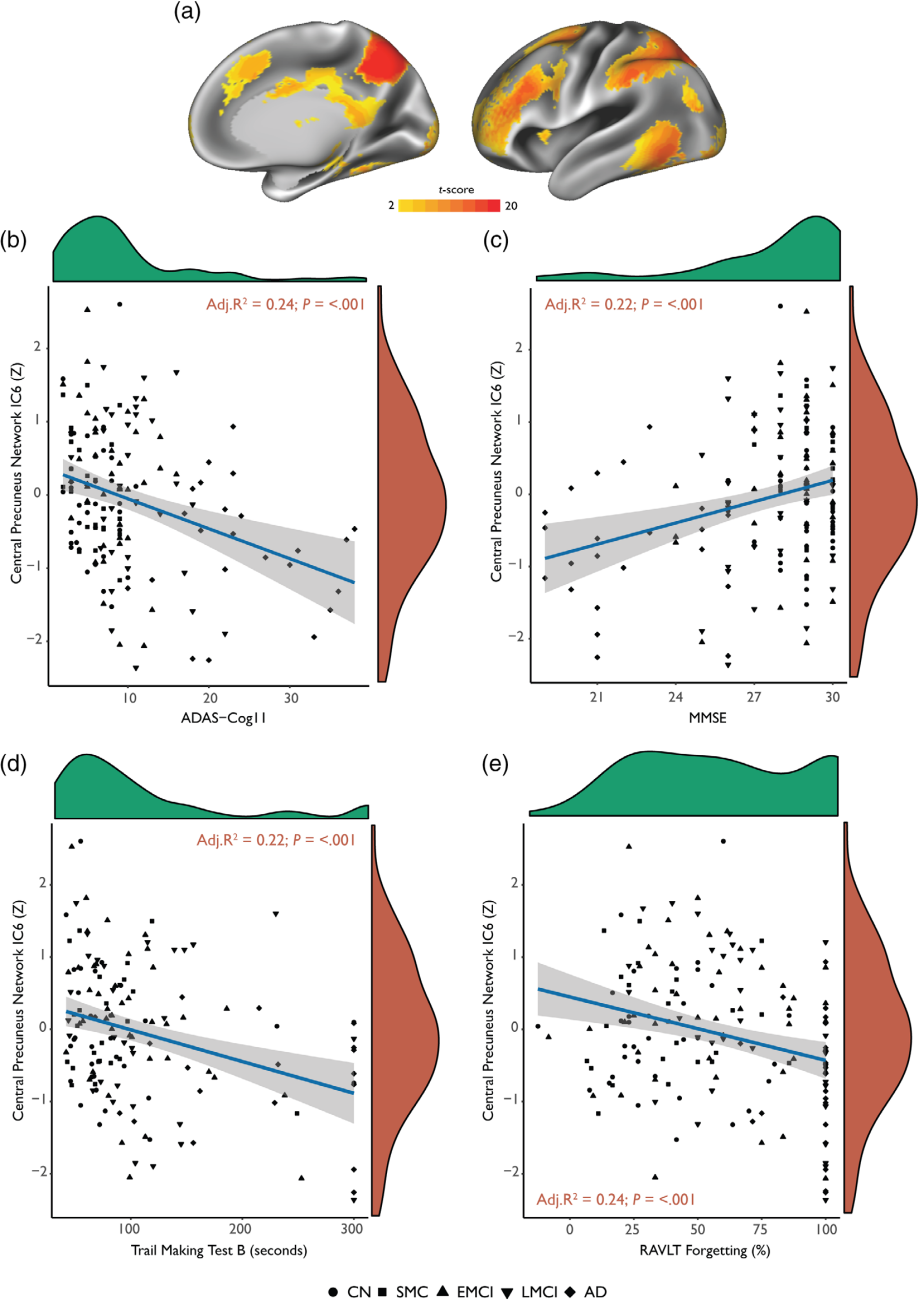
Functional connectivity of the central precuneus brain network is related to memory deficits and executive dysfunction across the Alzheimer's disease spectrum (*N* = 155). (a) Spatial map of the central precuneus “cognitive/associative” whole‐brain network is displayed on left lateral and right medial hemispheres. Functional connectivity of this network is plotted against (b) the 11‐item Alzheimer's disease assessment scale‐cognitive subscale (ADAS‐cog11) scores, (c) mini‐mental‐state examination (MMSE) scores, (d) trail making test B scores and (e) Rey auditory verbal learning test (RAVLT) forgetting scores expressed as percentages. The density distribution as marginal plots are displayed for cognitive variables in *green* and functional connectivity *Z*‐scores in *red*. Regression lines are shown in blue with 95% CIs (grey bands). Results displayed inset are from linear regression models. Age, gender, years of education and *APOE* ε4 genotype were considered as covariates in a stepwise fashion using Akaike Information Criterion minimisation. CN, cognitively normal; SMC, subjective memory complaints; EMCI, early mild cognitive impairment; LMCI, late mild cognitive impairment; AD, Alzheimer's disease

Results showed that weaker functional connectivity of the central precuneus was significantly associated with increasing disease severity measured using ADAS‐cog11 scores (Adj. *R* ^2^ = .22; *p* = <.001; Figure 5b). Weaker functional connectivity of the central precuneus was also significantly associated with lower MMSE scores (Adj. *R* ^2^ = .21; *p* = <.001; Figure 5c). A similar association was also observed with a measure of executive function, where lower central precuneus connectivity was significantly related to higher trail making test B scores (Figure 5d). A linear reduction in central precuneus functional connectivity was also associated with a linear increase in RAVLT percentage forgetting scores, suggesting that abnormal connectivity in the central precuneus network is also associated with verbal memory deficits (Figure 5e).

## 4 DISCUSSION

In the current study, we used a data‐driven approach to fractionate the PMC in the HCP dataset and assess how its distinct patterns of functional connectivity may be selectively vulnerable to network dysfunction in AD using the ADNI rsfMRI dataset. Using rsfMRI and multivariate analysis techniques a number of interesting findings emerged from this study. Firstly, the PMC was found to be functionally heterogeneous revealing a complex network architecture composed of discrete functional modules and distinct patterns of connectivity to widespread brain regions. Secondly, functional connectivity was not uniformly affected in AD, but rather selectively impacted the dorsal PCC and central precuneus, resulting in network failures that were associated with amyloid burden and volumetric hippocampal loss. Since the central precuneus has been previously implicated as an integrative hub for higher‐order cognitive processing, we investigated its patterns of functional connectivity across the AD pathological spectrum—defined here as a scale ranging from CN participants and participants with SMC, through to those with different stages of MCI, and patients diagnosed with AD. This investigation revealed that diminished functional connectivity of the central precuneus was related to clinical disease severity, as well as specific deficits in memory and executive function, showing that network aberrations in this region may underlie specific cognitive abnormalities in AD.

### 4.1 PMC possesses distinct functional modules with independent contributions from distributed networks

Previous evidence suggests that the PMC represents a critical gateway for information processing, acting as an interconnecting hub for converging information across segregated processing pathways (Parvizi et al., 2006; Vogt & Laureys, 2005). Functional connectivity analyses reveal that the PMC has a complex functional organisation and is highly functionally heterogenous (Dastjerdi et al., 2011; Margulies et al., 2009). It is also cytoarchitectonically distinct, with functional dissociation into a dorsal region showing strong connectivity patterns with multiple intrinsic networks and a ventral region that is highly integrated with the DMN at rest (Leech et al., 2011). Our work using two independent datasets to investigate the functional architecture of the PMC is consistent with these findings, showing that discrete functional modules of the PMC are associated with multiple large‐scale networks. More importantly, our parcellations show that the PCC possesses an extensive pattern of functional connectivity converging information across several networks involved in attentional control and cognition. The precuneus was also found to be highly functionally subspecialised with discrete subdivisions demarcating a broadly anterior–posterior functional divergence (S. Zhang & Li, 2012). This is consistent with previous anatomical and rsfMRI connectivity‐based parcellation studies of the precuneus demonstrating discrete functional roles for an anterior subdivision (executive), a central subdivision (cognitive/associative), and a posterior subdivision (visual information processing; Margulies et al., 2009). Finally, the RSC was delineated into two subdivisions underpinning a core network of brain regions known to be involved in a broad range of cognitive functions, including episodic memory, as well as navigation and future planning (Vann, Aggleton, & Maguire, 2009). Despite the complex functional organisation and high metabolic demands of the PMC, it is abnormally and preferentially affected by underlying neurodegenerative pathologies (Buckner et al., 2009; Seeley, Crawford, Zhou, Miller, & Greicius, 2009). However, little is known about how the distinct connectivity profiles of such a functionally heterogeneous association hub contribute to the network failures widely reported in AD.

### 4.2 Functional connectivity of the PMC is selectively impacted in AD

Having demonstrated that neural signals within the PMC are functionally discrete that reflect independent contributions from different networks, we next sought to determine the functional connectivity differences in AD using the ADNI dataset. For the PCC, we observed decreased functional connectivity patterns in the left ventral PCC and dorsal PCC subdivisions in AD patients compared to CN participants. Disruption of the ventral PCC network is consistent with previous studies showing a progressive breakdown of connections with the PCC and hippocampus in AD (Villain et al., 2010; Zhou et al., 2008), and previous findings highlighting the relationship between cingulum bundle atrophy and subsequent PCC hypometabolism (Villain et al., 2008). Moreover, these functional disruptions have been described in relation to an early and restricted involvement of the ventral PCC network, followed by subsequent cascades to the dorsal PCC at the AD dementia stage (Mutlu et al., 2016). However, we found the functional connectivity of the dorsal PCC was more prominently affected in AD, which is in accordance with a recent study showing that functional connectivity alterations originated in the dorsal PCC and later expanded to the ventral PCC region in severe AD cases (Wu et al., 2016). Recent work has also shown the dorsal PCC to be an integrative nexus of cortical connectivity responsible for modulating cognitive control processes (Leech et al., 2012). It is therefore plausible that the integrative network architecture, synchronous neural activity and region‐specific high information processing loads of the dorsal PCC may partly explain early episodic memory deficits in AD.

For the precuneus, we also observed functional connectivity decreases for the anterior and central subdivisions in AD patients. Altered precuneus functional connectivity has been extensively reported in AD (Binnewijzend et al., 2012; Damoiseaux, Prater, Miller, & Greicius, 2012; L. Wang et al., 2006). The precuneus has also been implicated in high‐level cognitive functions including episodic memory retrieval (Cavanna & Trimble, 2006; S. Zhang & Li, 2012) and previous neuroimaging findings have found disruptions of the precuneus to be associated with memory dysfunctions and visuospatial abnormalities in AD (D. Jones et al., 2011; Karas et al., 2007). It has been suggested that these abnormalities may underpin a precuneus‐hippocampal disconnection, and functional reductions in connected regions to the anterior cingulate (Sheline et al., 2010). Taken together, our findings concur with previous studies showing that different subregions of the PMC exhibit a differential vulnerability to AD (Petrella, Prince, Wang, Hellegers, & Doraiswamy, 2007; Wu et al., 2016; Xia et al., 2014).

The PMC is known to be one of the earlier regions to be preferentially affected by the neurodegenerative mechanisms in AD (Buckner et al., 2005; Klunk et al., 2004). Network failures in the dorsal PCC and central precuneus were found to be strongly associated with amyloid burden and volumetric hippocampal loss. Evidence suggests that neurodegenerative diseases may preferentially target functional networks of highly integrated association regions causing proteinopathies to topographically spread across synaptic convergence zones (Raj, Kuceyeski, & Weiner, 2012; Seeley et al., 2009; Warren et al., 2013). Furthermore, neurodegeneration in hippocampal regions has been postulated to disrupt network homeostasis (Jones et al., 2016) and a preferential accumulation of amyloid in hub regions has been linked to the disintegration of the DMN (Mormino et al., 2011; Palmqvist et al., 2017). However, there still remains a considerable debate as to whether widespread network dysfunctions in highly integrated regions play an aetiological role in the pathogenic spread of disease pathology or whether such aberrant network processes are secondary to degenerative insults (Jacobs et al., 2018).

### 4.3 Diminished functional connectivity of the central precuneus is associated with cognitive abnormalities across the AD spectrum

Despite the importance of the precuneus as a nexus for memory function in AD, it is relatively unknown how its underlying network properties become affected by AD and progress during different stages of the pathophysiological spectrum. Our findings demonstrate that the strength of functional connectivity in the central precuneus subdivision is associated with clinical measures of disease progression across the AD spectrum, suggesting that functional abnormalities in this region may be apparent prior to clinical symptoms. Previously, research has also shown that DMN abnormalities are widely evident in MCI patients and can be used to distinguish some that undergo cognitive decline and conversion to AD from those that remain clinically stable (Petrella, Sheldon, Prince, Calhoun, & Doraiswamy, 2011). Abnormal functional connectivity of the precuneus has also been reported in CN persons with elevated levels of amyloid (Drzezga et al., 2011), as well as CN *APOE* ε4 carriers without preclinical amyloid deposition (Sheline et al., 2010). We further show that reduced functional connectivity of the central precuneus is related to memory and executive abnormalities across the AD spectrum. This finding may suggest that cascading network failures in the central precuneus underpin the cognitive manifestations caused by the chronic effects of AD. A number of prior neuroimaging studies have also shown heightened activation of the precuneus during episodic and autobiographical memory tasks providing critical insights into the networks involvement in regulating cognition (Bzdok et al., 2015; Cavanna & Trimble, 2006; Margulies et al., 2009).

### 4.4 Limitations and future directions

In light of several interesting findings from this study, some potential limitations should be taken into consideration. In the ADNI dataset, rsfMRI scans are known to contain “pencilling” artefacts in part of the left lateral frontal lobe and have been observed to decrease functional connectivity in that region (Jones et al., 2016). Since we were uncertain as to how this may affect our analysis, we took all necessary precautions to avoid this region in our functional connectivity comparisons. Inherent limitations in fMRI scans obtained from the ADNI dataset could also be explained by the multi‐centre design of the study. Although considerable efforts in ADNI were taken to harmonise acquisition protocols across different study sites, we cannot exclude the possibility that some differences in acquisition may have remained.

Future explorations should also consider the recently described Human Connectome Project in Aging (HCP‐A) to elucidate how the functional connectivity of posterior midline regions of the cortex vary from the spectrum of normal ageing and neurodegenerative disease (Bookheimer et al., 2019). It may also be argued that a finer and more detailed organisation of the PMC can be unravelled when intrinsic connectivity is studied within an individual (Laumann et al., 2015). Recent evidence has suggested that the DMN comprises multiple parallel interdigitated networks that show specialisation across juxtaposed regions (Braga & Buckner, 2017). Future studies aiming to understand how these networks are modulated to control information processing may provide general insights into cognitive control and their dysfunction in neurodegenerative illnesses.

## 5 CONCLUSION

The functional organisation of the PMC uncovers distinct functional forms in local patterns of connectivity that are coupled to multiple large distributed networks in the brain. Analysing the connectivity profiles of these networks in AD reveals selective and prominent network disruptions of the dorsal PCC and central precuneus whose functional connectivity patterns are linked with amyloid burden and volumetric hippocampal loss. Across the entire AD pathological spectrum, diminished functional connectivity of the central precuneus is associated with disease severity and specific cognitive impairments, highlighting the relationship between network disintegration in this region and subsequent cognitive manifestations. Our findings accentuate the importance of a differential functional vulnerability of the PMC, showing that distinct functional abnormalities in this hub region are related to the pathological and cognitive manifestations of AD.

## CONFLICT OF INTEREST

The authors in this study report no conflicts of interest.

## Supporting information


**Data S1** Supplementary material.

**Figure S1** Reproducibility analysis of the ideal number of ICA components for subdividing the posteromedial cortex.

**Table S1** Complete list of subjects with corresponding ID's for resting‐state functional MRI and MPRAGE anatomical scans used in the study (*N* = 155).

## Data Availability

The data that support the findings of this study are available from the corresponding author upon reasonable request.
