# Supplementary material for: The heterogeneous functional architecture of the posteromedial cortex is associated with selective functional connectivity differences in Alzheimer's disease
Source: Hum Brain Mapp. 2019 Dec 19;41(6):1557–72. doi: 10.1002/hbm.24894 (PMC7268042; doi:10.1002/hbm.24894)
Supplement: Supplementary file 2 — Figure S1 Reproducibility analysis of the ideal number of ICA components for subdividing the posteromedial cortex. [file HBM-41-1557-s002.docx]

| 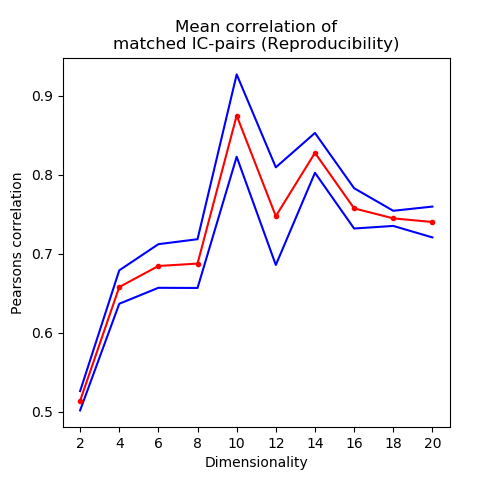  **Supplementary figure 1 reproducibility analysis of the ideal number of ICA components for subdividing the posteromedial cortex.** ﻿The ideal number of ICA components to use for fractionating the PMC were decided from the dimensionality-reproducibility curve (red = mean, blue = 95% CI’s), where the 10 component ICA was the global maximum. Therefore, the 10 component ICA analysis was chosen as the ideal number of components to subdivide the PMC into its distinct subdivisions. |
| --- |
