# Supplementary material for: The heterogeneous functional architecture of the posteromedial cortex is associated with selective functional connectivity differences in Alzheimer's disease
Source: Hum Brain Mapp. 2019 Dec 19;41(6):1557–72. doi: 10.1002/hbm.24894 (PMC7268042; doi:10.1002/hbm.24894)
Supplement: Supplementary file 3 — Table S1 Complete list of subjects with corresponding ID's for resting‐state functional MRI and MPRAGE anatomical scans used in the study (N = 155). [file HBM-41-1557-s003.docx]

**Supplementary Table 1**: Complete list of subjects with corresponding ID’s for resting-state functional MRI and MPRAGE anatomical scans used in the study (*N*=155)

| \| **RID** \| **Image ID**  **fMRI** \| **Image ID MPRAGE** \| \| --- \| --- \| --- \| \| 002_S_0295 \| 238623 \| 238627 \| \| 002_S_0413 \| 240811 \| 240812 \| \| 002_S_0685 \| 243902 \| 243898 \| \| 002_S_0729 \| 251176 \| 251177 \| \| 002_S_1155 \| 274147 \| 274154 \| \| 002_S_1261 \| 286519 \| 286516 \| \| 002_S_1280 \| 287493 \| 287487 \| \| 002_S_2010 \| 243875 \| 243872 \| \| 002_S_2043 \| 256635 \| 256637 \| \| 002_S_2073 \| 257487 \| 257483 \| \| 002_S_4171 \| 249536 \| 249535 \| \| 002_S_4213 \| 254581 \| 254582 \| \| 002_S_4225 \| 257271 \| 257270 \| \| 002_S_4229 \| 257477 \| 257476 \| \| 002_S_4237 \| 258448 \| 258443 \| \| 002_S_4264 \| 259806 \| 259796 \| \| 002_S_4270 \| 260580 \| 260581 \| \| 002_S_4447 \| 278818 \| 278815 \| \| 002_S_4473 \| 283879 \| 283874 \| \| 002_S_4521 \| 285812 \| 285808 \| \| 002_S_4654 \| 296366 \| 296365 \| \| 002_S_4746 \| 306889 \| 306887 \| \| 002_S_4799 \| 311921 \| 311922 \| \| 002_S_5018 \| 346237 \| 346242 \| \| 002_S_5178 \| 372853 \| 372851 \| \| 002_S_5230 \| 378490 \| 378487 \| \| 002_S_5256 \| 380899 \| 380901 \| \| 006_S_4150 \| 249406 \| 249403 \| \| 006_S_4153 \| 248516 \| 248517 \| \| 006_S_4192 \| 258605 \| 258594 \| \| 006_S_4346 \| 266129 \| 266138 \| \| 006_S_4357 \| 268917 \| 268919 \| \| 006_S_4363 \| 269256 \| 269254 \| \| 006_S_4449 \| 279468 \| 279470 \| \| 006_S_4485 \| 281887 \| 281882 \| \| 006_S_4515 \| 283264 \| 283271 \| \| 006_S_4679 \| 307555 \| 307551 \| \| 006_S_4713 \| 303731 \| 303729 \| \| 006_S_4867 \| 322000 \| 322012 \| \| 006_S_4960 \| 339123 \| 339124 \| \| 006_S_5153 \| 368159 \| 368152 \| \| 010_S_4442 \| 283913 \| 283915 \| \| 012_S_4026 \| 238542 \| 238532 \| \| 012_S_4094 \| 243880 \| 243881 \| \| 012_S_4128 \| 248233 \| 248234 \| \| 012_S_4188 \| 255318 \| 255322 \| \| 012_S_4643 \| 297689 \| 297693 \| \| 012_S_4849 \| 319132 \| 319138 \| \| 012_S_4987 \| 343905 \| 343906 \| \| 012_S_5121 \| 363549 \| 363544 \| \| 012_S_5157 \| 372896 \| 372891 \| \| 012_S_5195 \| 376798 \| 376803 \| \| 012_S_5213 \| 381905 \| 381899 \| \| 013_S_1186 \| 287005 \| 287002 \| \| 013_S_2324 \| 305412 \| 305410 \| \| 013_S_2389 \| 339828 \| 339821 \| \| 013_S_4268 \| 264416 \| 264420 \| \| 013_S_4395 \| 278367 \| 278368 \| \| 013_S_4579 \| 296769 \| 296776 \| \| 013_S_4580 \| 296863 \| 296859 \| \| 013_S_4595 \| 300057 \| 300051 \| \| 013_S_4616 \| 300088 \| 300089 \| \| 013_S_4791 \| 337377 \| 337383 \| \| 013_S_4917 \| 332584 \| 330023 \| \| 013_S_4985 \| 339635 \| 339623 \| \| 013_S_5071 \| 360323 \| 360324 \| \| 013_S_5137 \| 368206 \| 368204 \| \| 013_S_5171 \| 374972 \| 374978 \| \| 018_S_2133 \| 280337 \| 280329 \| \| 018_S_2155 \| 274112 \| 274113 \| \| 018_S_2180 \| 281032 \| 281027 \| \| 018_S_4257 \| 262078 \| 262076 \| \| 018_S_4349 \| 266634 \| 266625 \| \| 018_S_4399 \| 272223 \| 272231 \| \| 018_S_4400 \| 273503 \| 273504 \| \| 018_S_4597 \| 290305 \| 290304 \| \| 018_S_4733 \| 306073 \| 306069 \| \| 018_S_4868 \| 319541 \| 319536 \| \| 018_S_4889 \| 323221 \| 323219 \| \| 018_S_5240 \| 382187 \| 382181 \| \| 018_S_5262 \| 385115 \| 385124 \| \| 019_S_4252 \| 258955 \| 258947 \| \| 019_S_4285 \| 260905 \| 260913 \| \| 019_S_4293 \| 261984 \| 261983 \| \| 019_S_4367 \| 269279 \| 269273 \| \| 019_S_4477 \| 280778 \| 280783 \| \| 019_S_4548 \| 286477 \| 286475 \| \| 019_S_4680 \| 300529 \| 300530 \| \| 019_S_4835 \| 315850 \| 315857 \| \| 019_S_5012 \| 343912 \| 343916 \| \| 019_S_5242 \| 379089 \| 379092 \| \| 031_S_2018 \| 242177 \| 242175 \| \| 031_S_2022 \| 247662 \| 247671 \| \| 031_S_2233 \| 275532 \| 275535 \| \| 031_S_4024 \| 228872 \| 228879 \| \| 031_S_4029 \| 229511 \| 229510 \| \| 031_S_4032 \| 234917 \| 234922 \| \| 031_S_4042 \| 235238 \| 235235 \| \| 031_S_4149 \| 249144 \| 249147 \| \| 031_S_4194 \| 255284 \| 255288 \| \| 031_S_4203 \| 255309 \| 255316 \| \| 031_S_4218 \| 255986 \| 255978 \| \| 031_S_4474 \| 280365 \| 280369 \| \| 031_S_4476 \| 281149 \| 281143 \| \| 031_S_4496 \| 282646 \| 282638 \| \| 031_S_4590 \| 290644 \| 290642 \| \| 031_S_4947 \| 339436 \| 339439 \| \| 053_S_0919 \| 354654 \| 354657 \| \| 053_S_2357 \| 305210 \| 305212 \| \| 053_S_2396 \| 319578 \| 319577 \| \| 053_S_4557 \| 289331 \| 289333 \| \| 053_S_4578 \| 290815 \| 290814 \| \| 053_S_4661 \| 306672 \| 306676 \| \| 053_S_5070 \| 357475 \| 357474 \| \| 053_S_5202 \| 375698 \| 375693 \| \| 053_S_5208 \| 376933 \| 376939 \| \| 053_S_5272 \| 385101 \| 385108 \| \| 053_S_5287 \| 389318 \| 389320 \| \| 053_S_5296 \| 401123 \| 401126 \| \| 100_S_4512 \| 298266 \| 298265 \| \| 100_S_4556 \| 300841 \| 300845 \| \| 100_S_5091 \| 362420 \| 362425 \| \| 100_S_5096 \| 364799 \| 364804 \| \| 100_S_5102 \| 365276 \| 365269 \| \| 100_S_5106 \| 368413 \| 368412 \| \| 100_S_5280 \| 387121 \| 387122 \| \| 130_S_2373 \| 302589 \| 302592 \| \| 130_S_2403 \| 341127 \| 341131 \| \| 130_S_4250 \| 259691 \| 259694 \| \| 130_S_4294 \| 267894 \| 267899 \| \| 130_S_4343 \| 266208 \| 266217 \| \| 130_S_4352 \| 267713 \| 267711 \| \| 130_S_4405 \| 279103 \| 279102 \| \| 130_S_4415 \| 278493 \| 278496 \| \| 130_S_4417 \| 279181 \| 279186 \| \| 130_S_4542 \| 287650 \| 287648 \| \| 130_S_4641 \| 295969 \| 295961 \| \| 130_S_4730 \| 306375 \| 306384 \| \| 130_S_4817 \| 314327 \| 314330 \| \| 130_S_4883 \| 323163 \| 323159 \| \| 130_S_4925 \| 337131 \| 337132 \| \| 130_S_4971 \| 342326 \| 342338 \| \| 130_S_4982 \| 341793 \| 341787 \| \| 130_S_4984 \| 342278 \| 342274 \| \| 130_S_4990 \| 342915 \| 342911 \| \| 130_S_4997 \| 347402 \| 347410 \| \| 130_S_5006 \| 348491 \| 348490 \| \| 130_S_5059 \| 358777 \| 358776 \| \| 130_S_5142 \| 368288 \| 368283 \| \| 130_S_5175 \| 374299 \| 374298 \| \| 130_S_5258 \| 381475 \| 381457 \| \| 131_S_5148 \| 367728 \| 367769 \| \| 136_S_4269 \| 264214 \| 264215 \| \| 136_S_4433 \| 279084 \| 278511 \| \| 136_S_4517 \| 293995 \| 294000 \| |
| --- | --- | --- | --- | --- | --- | --- | --- | --- | --- | --- | --- | --- | --- | --- | --- | --- | --- | --- | --- | --- | --- | --- | --- | --- | --- | --- | --- | --- | --- | --- | --- | --- | --- | --- | --- | --- | --- | --- | --- | --- | --- | --- | --- | --- | --- | --- | --- | --- | --- | --- | --- | --- | --- | --- | --- | --- | --- | --- | --- | --- | --- | --- | --- | --- | --- | --- | --- | --- | --- | --- | --- | --- | --- | --- | --- | --- | --- | --- | --- | --- | --- | --- | --- | --- | --- | --- | --- | --- | --- | --- | --- | --- | --- | --- | --- | --- | --- | --- | --- | --- | --- | --- | --- | --- | --- | --- | --- | --- | --- | --- | --- | --- | --- | --- | --- | --- | --- | --- | --- | --- | --- | --- | --- | --- | --- | --- | --- | --- | --- | --- | --- | --- | --- | --- | --- | --- | --- | --- | --- | --- | --- | --- | --- | --- | --- | --- | --- | --- | --- | --- | --- | --- | --- | --- | --- | --- | --- | --- | --- | --- | --- | --- | --- | --- | --- | --- | --- | --- | --- | --- | --- | --- | --- | --- | --- | --- | --- | --- | --- | --- | --- | --- | --- | --- | --- | --- | --- | --- | --- | --- | --- | --- | --- | --- | --- | --- | --- | --- | --- | --- | --- | --- | --- | --- | --- | --- | --- | --- | --- | --- | --- | --- | --- | --- | --- | --- | --- | --- | --- | --- | --- | --- | --- | --- | --- | --- | --- | --- | --- | --- | --- | --- | --- | --- | --- | --- | --- | --- | --- | --- | --- | --- | --- | --- | --- | --- | --- | --- | --- | --- | --- | --- | --- | --- | --- | --- | --- | --- | --- | --- | --- | --- | --- | --- | --- | --- | --- | --- | --- | --- | --- | --- | --- | --- | --- | --- | --- | --- | --- | --- | --- | --- | --- | --- | --- | --- | --- | --- | --- | --- | --- | --- | --- | --- | --- | --- | --- | --- | --- | --- | --- | --- | --- | --- | --- | --- | --- | --- | --- | --- | --- | --- | --- | --- | --- | --- | --- | --- | --- | --- | --- | --- | --- | --- | --- | --- | --- | --- | --- | --- | --- | --- | --- | --- | --- | --- | --- | --- | --- | --- | --- | --- | --- | --- | --- | --- | --- | --- | --- | --- | --- | --- | --- | --- | --- | --- | --- | --- | --- | --- | --- | --- | --- | --- | --- | --- | --- | --- | --- | --- | --- | --- | --- | --- | --- | --- | --- | --- | --- | --- | --- | --- | --- | --- | --- | --- | --- | --- | --- | --- | --- | --- | --- | --- | --- | --- | --- | --- | --- | --- | --- | --- | --- | --- | --- | --- | --- | --- | --- | --- | --- | --- | --- | --- | --- | --- | --- | --- | --- | --- | --- | --- | --- | --- | --- | --- | --- | --- | --- | --- | --- | --- | --- | --- | --- | --- | --- | --- | --- | --- | --- | --- | --- | --- | --- | --- | --- | --- | --- | --- | --- | --- | --- | --- | --- | --- | --- | --- | --- | --- | --- | --- | --- | --- | --- | --- | --- | --- |
